# COVID-19 and Health Disparities: Structural Evil Unmasked

**DOI:** 10.5334/aogh.3225

**Published:** 2021-04-01

**Authors:** Philip J. Landrigan, Lilian Ferrer, James Keenan

**Affiliations:** 1 Program for Global Public Health and the Common Good, Boston College, US; 2 Pontificia Universidad Católica de Chile, CL; 3 Jesuit Institute, Boston College, US

## Abstract

**Background:**

Incidence and mortality from COVID-19 are starkly elevated in poor, minority and marginalized communities. These differences reflect longstanding disparities in income, housing, air quality, preexisting health status, legal protections, and access to health care. The COVID-19 pandemic and its economic consequences have made these ancient disparities plainly visible.

**Methodology:**

As scholars in Catholic research universities committed to advancing both scientific knowledge and social justice, we examined these disparities through the lenses of both epidemiology and ethics.

**Findings:**

We see these widening disparities as not only as threats to human health, societal stability, and planetary health, but also as moral wrongs - outward manifestations of unrecognized privilege and greed. They are the concrete consequences of policies that promote structural violence and institutionalize racism.

**Recommendations:**

We encourage governments to take the following three scientific and ethical justified actions to reduce disparities, prevent future pandemics, and advance the common good: (1) Invest in public health systems; (2) Reduce economic inequities by making health care affordable to all; providing education, including early education, to all children; strengthening environmental and occupational safeguards; and creating more just tax structures; *and* (3) Preserve our Common Home, the small blue planet on which we all live.

Incidence and mortality from COVID-19 are much higher in poor, minority and marginalized communities than in other sectors of society [1,2]. These differences reflect longstanding disparities in income, housing, air quality, preexisting health status, legal protections, and access to health care [3]. These disparities are ancient, and in the United States many had their origins in slavery [4], but the COVID-19 pandemic and the economic devastation it has wrought have brought them into the open and made them clearly visible [5].

In many countries, disparities are widening. Political leaders are overtly encouraging racism and fostering inequities based on gender, race, class, immigration status and economics [6]. Weakening of public health systems, reductions in access to affordable health care, and steep increases in drug prices disproportionately affect the poor and further widen disparities [7]. Rollbacks of environmental and occupational safeguards result in disproportionate exposures of low-income communities and industrial workers to pollution, increased rates of environmentally and occupationally related disease, and increased risk of death [8,9]. Regressive tax policies reduce taxation on the wealthy while diminishing real income for working families and the poor [10]. Reckless exploitation of the planet’s resources and unending increases in the combustion of fossil fuels enrich the few while destroying the health, the livelihoods, and the dignity of the many [11].

As scholars in Catholic research universities committed to advancing both scientific knowledge and social justice, we are compelled to call out these inequities and to examine them not only with the tools of science but also through the lens of ethics and morality.

Thus, we see the widening disparities that have become so painfully apparent in the COVID-19 pandemic as not only threats to human health, societal stability, and planetary health, but also as moral wrongs – outward manifestations of unrecognized privilege and greed. This ethical assessment opens up new strategies for eliminating disparities and advancing the common good, e.g., the formation of partnerships between scientists, ethicists and communities of faith. It also offers new metrics for assessing our progress toward these goals, e.g., the collection of national statistics on economic inequality and rates of pollution-related disease.

Philosophical and theological traditions summon us to an ethical apprehension of reality, in which we are called to recognize that human well-being is dependent on the community and that each member of society must equitably have access to that which we call the common good. Moreover, every moral system asserts an interest in the worth, dignity, and rights of both the individual and the community and relies on the principle of equity to measure the means for attaining human flourishing through attainment of the common good.

A further recognition that has grown in the past decade among philosophers, ethicists, theologians and scientists is that enhancement of the well-being of the human community cannot be achieved at the expense of the health of the planet and its ecosystems. Ethics then serves as a reminder that regard for the human and planetary health cannot be separated and that the pursuit of human well-being must be accountable, transparent, just, and sustainable [12].

The disparities we see today are the concrete consequences of long-standing policies that promote structural violence and institutionalize racism. These policies harden boundaries and deepen differences. They contravene the core commandment of Christianity to, “Love thy neighbor as thyself.” They fly in the face of Pope Francis’ call for a preferential option for the poor [11]. In the United States, accounts of these unaddressed, oppressive expressions of privileged power are found in Reinhold Niebuhr’s *Moral Man, Immoral Society* and more recently in Isabel Wilkerson’s *Caste* [13,14]. Most other nations offer similar narratives. We have known these tales for too long.

Yet in this pandemic, we also see signs of hope. The selfless heroism of health workers, first responders and transit workers provide shining examples of the good of which people are capable. The coming together of millions of people in countries across the globe have to denounce hatred and racism is a powerful affirmation of human dignity. Pandemic-related improvements in air quality have reduced pollution-related disease and show that clean air is possible [15]; they enable us to imagine a world in which improvements in air quality are permanent and most energy is produced from non-polluting, renewable fuels. The successes of countries such as Georgia, Germany, Rwanda, South Korea, Switzerland, and New Zealand in containing the pandemic by investing in public health systems, deliberately addressing inequities, and assisting the vulnerable demonstrate that scientifically sound and morally responsible public policy is not only possible, but also highly effective.

A key question is how to build on this hope. How do we seize the moment of opportunity presented by the COVID-19 pandemic to make our societies just, sustainable, and resilient – societies that prioritize equity, health, well-being, and happiness over endless growth and widening disparities? How do we incorporate the ethical call for solidarity among all people into the fabric of our societies and turn this summons into a daily realty? [16].

Three scientifically sound and morally justified actions that will build on this hope and reduce disparities are the following:

- **Invest in public health systems at every level – international, national state/provincial and local.** All countries need to learn from the experience of the countries that entered this pandemic with robust public health systems, acted decisively, and successfully contained COVID-19. Just as SARS, MERS, Ebola, Zika and chikungunya have emerged in the past two decades, new diseases will almost certainly appear in the years ahead. Both science and ethics demand a sustained investment in health systems precisely because emerging diseases further alienate and disproportionately harm those whose rights are already insufficiently protected.
- **Reduce economic inequity by making health care affordable to all; providing education, including early education, to all children; strengthening environmental and occupational safeguards; and creating more just tax structures.** These policies, when carefully designed and implemented, will lift people from poverty, increase societal stability, build community and engender hope [7,10].
- **Preserve the health and well-being of all people in current and future generations by protecting the small blue planet on which we all live – our Common Home** [11]. All countries must recommit to the Paris Climate Accords and reduce greenhouse gas emissions. This is both a scientific and a moral imperative. Countries need to accelerate their transition to non-polluting, renewable sources of energy by investing in clean energy research, increasing incentives for wind and solar power and ending all governmental subsidies and tax breaks for fossil fuels [15]. Inasmuch as the pursuit of the common good through equity is the long-standing goal of ethics, rich countries need to step up, behave generously and assume more responsibility to assist low-income and middle-income countries in this energy transition.

At a more fundamental level, all of us need to reexamine our commitment to the common good and our awareness of the dignity of all people. In our daily lives, we need to balance the exercise of our individual rights against our responsibilities to our communities and to the vulnerable among us. We need to be aware of both the danger of setting ourselves apart from others and the strengths of solidarity. We must heed always the moral and ethical imperative to protect and better incorporate the poor and the weak. The actions we take to rebuild public health systems, reduce disparities and preserve our Common Home are all justified by sound science. But at the same time, they are ethical means to promote our shared commitment to the common good and human solidarity [16].

Elected officials and leaders of governments have particular ethical and moral responsibilities in this time of COVID-19. They have the obligation to strive, even in the face of great opposition to advance the health, well-being, and dignity of all people and to break down the racial, ethnic and economic barriers that perpetuate injustice and inequity. They need to recognize the grave dangers of alienation and the risks of becoming self-promoting, isolated nationalistic states that ignore the rights of others. They need to develop an ethics of vulnerability and an ability to work generously with other leaders in sustainable ways to build global cooperation. They must develop and abide by international standards and protocols regarding migration and refugee status. They are morally and ethically bound to dismantle structures that use race, tribe, gender and caste biases to thwart the principle of equity.

The pain and suffering we have seen in the COVD-19 pandemic remind us of basic moral truths. Both science and ethics have unveiled evil realities that we have ignored for far too long. At the same time, the hope and the altruism we see in this pandemic remind us of who we can become and of how a solidarity among all people can emerge from within contemporary society [16]. They remind us that we can indeed reorient our moral compass, recommit ourselves to advancing the common good, and create a better, more hopeful life for all.
